# Oxygen-Tolerant Ab Initio Emulsion ATRP Driven by Red or Near-Infrared Light

**DOI:** 10.1021/acs.macromol.6c01379

**Published:** 2026-06-22

**Authors:** Xiaolei Hu, Kangping Liu, Krzysztof Matyjaszewski

**Affiliations:** Department of Chemistry, 6612 Carnegie Mellon University, Pittsburgh, Pennsylvania 15213, United States

## Abstract

Emulsion polymerization technique is widely used as a practical and greener approach to polymer synthesis. However, previous reversible deactivation radical polymerization (RDRP) in heterogeneous systems has mostly focused on microemulsion and miniemulsion, requiring a large amount of surfactant and/or vigorous homogenization. To expand the scope and enhance the practicality of RDRP in emulsion, we demonstrate a highly efficient and O_2_-tolerant ab initio emulsion atom transfer radical polymerization (ATRP), driven by red or near-infrared (NIR) light. This was facilitated using a water-soluble photocatalyst, methylene blue (MB^+^), a hydrophilic initiator, and an interfacial ion-pair catalyst. Excitation of MB^+^ under red/NIR light irradiation enabled efficient photoreduction of the ATRP deactivator and subsequent initiation in the aqueous phase, thereby mediating polymerization via a classic emulsion mechanism. The ab initio emulsion ATRP provided successful synthesis of polymers with controlled molar masses ranging from 10,000 to 600 000 g/mol and low dispersity (1.09 ≤ *Đ* ≤ 1.29), excellent chain-end fidelity, and facile temporal control.

## Graphical abstract

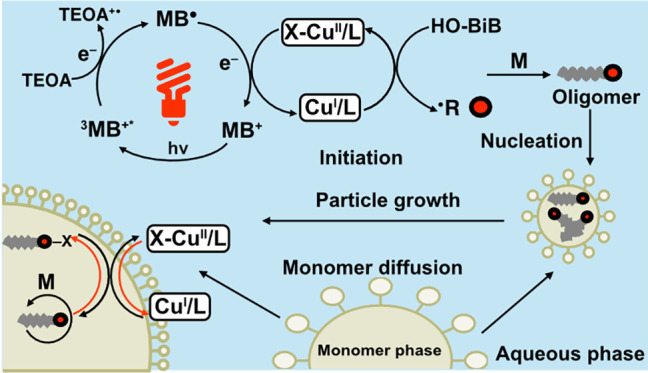

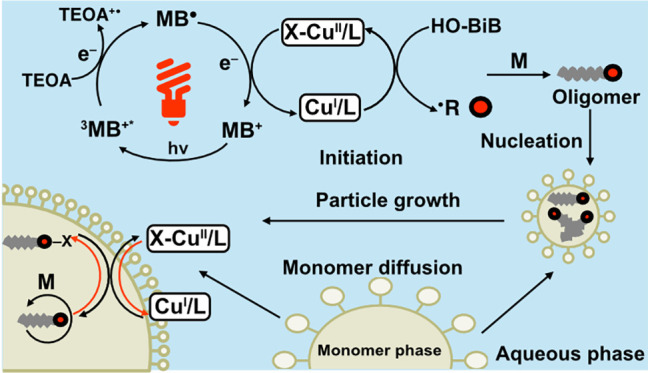

## Introduction

Emulsion polymerization is a cornerstone of modern industrial polymer production, offering high reaction rate, effective heat dissipation, and the synthesis of polymers in a greener aqueous environment.^1,2^ In recent years, reversible deactivation radical polymerization (RDRP) techniques, particularly atom transfer radical polymerization (ATRP) and reversible addition–fragmentation chain transfer (RAFT) polymerization, have been adapted to heterogeneous media to provide precise control over molecular weight, architecture, and functionality.^3–21^

Previous efforts on the RDRP in dispersed media include (inverse) microemulsion,^22,23^ miniemulsion,^24–32^ emulsion,^33–36^ and dispersion.^37–39^ However, microemulsion requires large amounts of surfactant to maintain thermodynamic stability, and miniemulsion demands vigorous homogenization (typically probe ultrasonication or microfluidization) to generate the initial submicron droplets, both of which significantly limit their practical scalability.^21^ Among these, ab initio emulsion is the most common and robust approach for industrial polymer synthesis because it utilizes low surfactant concentrations and eliminates the need for external homogenization. However, it remains rarely exploited in RDRP due to its mechanistic complexity: the polymerization requires aqueous initiation and nucleation, followed by monomer diffusion into growing polymer particles and propagation within the hydrophobic phase. For example, the ATRP catalyst [Cu^II^/L]^+^ (where L is an ATRP ligand) must “dynamically follow” the growing radicals as they transfer from the aqueous phase into the organic phase, making precise catalyst partitioning critical for maintaining controlled emulsion ATRP. To address this challenge, several strategies have been developed, including (i) a seeded emulsion approach, where initial microemulsion or miniemulsion ATRP encapsulates the hydrophobic catalyst into seed particles that are subsequently swelled with fresh monomer;^40^ (ii) the use of a phase-transfer catalyst, such as tetrabutylammonium bromide, to shuttle the catalyst between phases;^41^ and (iii) a surfactant-ligand design that immobilizes the Cu catalyst at the droplet interface.^42^ More recently, our group demonstrated that the interfacial ion-pair catalyst [Br–Cu^II^L^+^/DS^–^], formed by the binding of a cationic [Br–Cu^II^/L]^+^ complex with the anionic surfactant sodium dodecyl sulfate (SDS), enables true ab initio emulsion ATRP.^36^ This system was subsequently extended to photoinduced ATRP (photoATRP) under UV light irradiation, achieving temporal control and well-defined polymer architectures across a range of methacrylate monomers.^33^

Despite this progress, current classic emulsion ATRP still suffers from low efficiency and poor oxygen tolerance, necessitating a rigorous deoxygenation step. To address these limitations, we report a robust ab initio emulsion ATRP system driven by red and near-infrared (NIR) light by utilizing a water-soluble photocatalyst, methylene blue (MB^+^). Indeed, this effective photocatalyst has demonstrated robust ATRP in homogeneous^43^ and heterogeneous systems (i.e., both miniemulsion^32^ and inverse emulsion^23^). To transition from microemulsion and miniemulsion to a more practical ab initio emulsion, we replaced the hydrophobic initiators typically used in heterogeneous RDRP with the water-soluble initiator 2-hydroxyethyl α-bromoisobutyrate (HO-EBiB). As illustrated in Scheme 1, the process is initiated by dual catalysis in the aqueous phase, involving MB^+^ and the interfacial ion-pair catalyst [Br–Cu^II^L^+^/DS^–^]. Upon irradiation, the excited MB^+^ is reduced by triethanolamine (TEOA) to generate the MB radical species. This species subsequently reduces the [Br–Cu^II^/L]^+^ deactivator to form [Cu^I^/L]^+^ in the aqueous phase. The resulting activator reacts with the hydrophilic initiator HO-EBiB to generate initiating radicals in the water phase. These radicals initiate the propagation of dissolved monomer molecules, forming water-soluble oligomers. Once these oligomers reach a critical chain length, they are captured by monomer-swollen micelles. Following nucleation, the polymerization proceeds within the monomer-swollen particles, sustained by the continuous diffusion of monomer from the droplets through the aqueous phase. The interfacial ion-pair catalyst enables the ATRP equilibrium throughout the emulsion polymerization, thus achieving precise control over polymerization. This classic emulsion photoATRP proceeds without requiring prior homogenization and at surfactant concentrations as low as 2.3 wt % relative to the monomer, significantly lower than in traditional miniemulsion and microemulsion systems. Importantly, this long-wavelength dual-catalysis system by MB^+^ facilitates polymerization at ambient conditions without prior deoxygenation. This work represents a robust and industrially applicable emulsion technology, providing a facile and precise route for the synthesis of advanced polymeric materials.

**Scheme 1:**
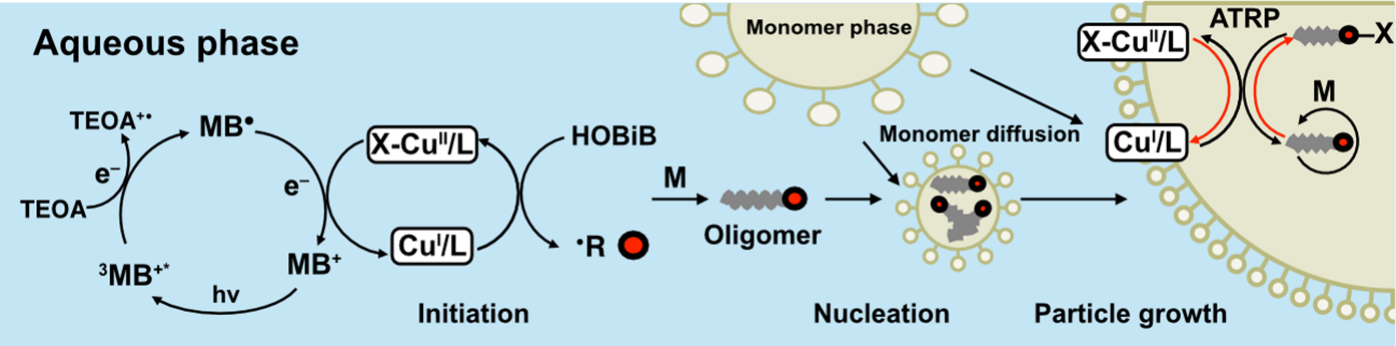
Proposed Mechanism for Red/NIR Light-Driven Ab Initio Emulsion PhotoATRP]

## Results and Discussion

### Polymerization Conditions

We began by confirming the proposed concept for ab initio photoATRP using *n*-butyl methacrylate (BMA) as the monomer and water as the dispersed medium (Figure S1). The dual catalytic system comprised a [X–Cu^II^/TPMA]^+^ complex as the deactivator, MB^+^ as the PC, and TEOA as the electron donor (ED). SDS was utilized not only as the surfactant but also to generate an ion-pair catalyst [X–Cu^II^L^+^/DS^–^] by binding to [X–Cu^II^/TPMA]^+^. This resulting catalyst could effectively mediate propagating radicals throughout the lifetime of the polymer chain, from initiation in the aqueous phase to nucleation and growth within latex nanoparticles (NPs),^33,36^ thus facilitating an efficient ATRP process in classic emulsion.^44^ Notably, in contrast to the oil-soluble initiators typically used in miniemulsion and microemulsion systems, the water-soluble initiator HO–EBiB was used to generate initiating radicals in the aqueous phase for the ab initio process. The polymerizations were performed in a one-dram vial under red LED (640 nm, 25 mW cm^–2^) irradiation without prior deoxygenation.

As shown in Entry 1 in Table 1, a successful polymerization was observed when all the reagents (i.e., MB^+^, HO–EBiB, CuBr_2_/TPMA, and TEOA) were used together, achieving a monomer conversion of 95.3% within 4.5 h. The resulting polymer exhibited well-defined MW and low dispersity (*M*_n,th_ = 68,000, *M*_n,abs_ = 72,100, *Đ* = 1.25), yielding monodisperse NPs with a size of 45 nm at [SDS] = 6.9 wt % (Figures S2-S3). Control experiments excluding the initiator led to uncontrolled free radical polymerization initiated by dual catalysis (Table 1, Entry 2). In addition, the absence of the MB^+^, ATRP deactivator, or TEOA resulted in negligible conversion due to the lack of a necessary component for dual catalysis (Table 1, Entries 3–5). The critical role of the water-soluble initiator was further highlighted by replacing HO-EBiB with a hydrophobic initiator, ethyl α-bromophenylacetate (EBPA). This substitution resulted in poorly controlled polymerization characterized by a shoulder peak at high molecular weight and significantly broader dispersity (Table 1, Entry 6). This was plausibly due to the partitioning of the hydrophobic initiator at the onset of polymerization, leading to heterogeneous initiation in both the aqueous and monomer phases rather than an ab initio mechanism. Additionally, DLS results revealed the presence of large aggregates (6.8 μm). These results highlight the critical role of the MB^+^/[Br–Cu^II^L^+^/DS^–^] interfacial catalytic system and water-soluble initiator for successful ab initio emulsion photoATRP.

**Table 1:** Optimization of Polymerization Conditions^a^

Entry	HO-EBiB (equiv)	MB^+^ (equiv)	CuBr_2_/L (equiv)	TEOA (equiv)	Conv.^b^ (%)	*M*_n,th_	*M*_n,app_ ^c^	*M*_n,abs_ ^d^	*Đ* ^c^	Z_ave_ ^e^ (nm)
1	1	0.025	0.1	0.6	95.3	68,000	66,200	72,100	1.25	46.7 ± 0.2
2	–	0.025	0.1	0.6	72.9	–	115,300	127,100	2.98	47.3 ± 0.1
3	1	–	0.1	0.6	1.5	–	–		–	–
4	1	0.025	–	0.6	2.2	–	–		–	–
5	1	0.025	0.1	–	1.2	–	–		–	–
6^f^	1	0.025	0.1	0.6	45.9	42,300	45,900	49,600	4.48	6838 ± 496

a Reaction conditions: [BMA]/[HO-EBiB]/[MB^+^]/[CuBr_2_/TPMA (1:1 molar ratio)]/[TEOA] = 500/X/X/X/X, [M] = 20 vol % of the total, [SDS] = 6.9 wt % relative to BMA, [NaBr] = 0.1 M, irradiated under red LED (640 nm, 25 mW cm^–2^) for 4.5 h in a one dram vial (diameter = 15 mm) with stirring.

b The monomer conversion was determined by gravimetric analysis.

c Molecular weight (*M*_n,app_) and dispersity (*Đ*) were determined by SEC analysis (THF as eluent) calibrated to polystyrene standards.

d The absolute molecular weight (*M*_n,abs_) was determined by Mark–Houwink calibration.^45,46^

e The average particle diameter (Z_avg_) was determined by DLS.

f EBPA was used instead of HO-EBiB.

We further explored the effect of surfactant concentration on ab initio photoATRP. As the SDS concentration was decreased from 6.9 to 4.6 and 2.3 wt % (Table S1), particle sizes increased from 45 to 64 nm. While the resulting emulsion maintained good control over MW and colloidal stability, the reduced surfactant loading caused a slight increase in polymer dispersity, plausibly due to a lower concentration of interfacial cocatalyst [X–Cu^II^L^+^/DS^–^] and surfactant available to stabilize the emulsion particles. Notably, well-defined polymer was obtained at only 2.3 wt % SDS without prior deoxygenation, significantly lower than in traditional microemulsion and miniemulsion systems. This highlights the key practical advantage of our approach for efficient and sustainable polymer synthesis.

### Kinetic Study

To evaluate the control of the ab initio emulsion photoATRP, the polymerization kinetics of BMA was investigated under NIR LED irradiation (740 nm, 20 mW cm^–2^) at an SDS concentration of 2.3 wt %. Under light irradiation, atom transfer from a hydrophilic initiator via dual catalysis generated radicals to initiate the polymerization of BMA dissolved in the aqueous phase, forming oligomers. These oligomers were subsequently captured by micelles after reaching a certain DP, and polymerization progressed as the micelles continued to grow using the monomer diffused from monomer droplets, eventually forming a stable emulsion latex. As shown in Figure S4, the reaction mixture gradually became turbid, and the monomer phase disappeared as polymerization progressed. The kinetic analysis revealed a short induction period of ca. 30 min, accounting for the consumption of O_2_ in the reaction mixture, followed by polymerization that achieved nearly quantitative monomer conversion within 8 h (Figure 1A). The absolute molecular weights (*M*_n,abs_) increased linearly as a function of monomer conversion, showing good agreement with the theoretical values (*M*_n,th_, solid line in Figure 1B). A slight increase in *Đ* at high conversion (1.15 ≤ *Đ* ≤ 1.56) was observed, which could be attributed to the fact that the surfactant concentration ([SDS] = 14.3 mM at 2.3 wt %) was only marginally above the critical micelle concentration (CMC) of SDS (∼8.3 mM at 25 °C),^47^ leaving insufficient free surfactant to stabilize the growing NPs. In addition, the monomodal SEC traces shifted toward the high molecular weight (MW) region (Figure 1C). Simultaneously, the evolution of the average particle size (Z_ave_) as a function of conversion revealed a steady increase from approximately 36 to 70 nm (Figure 1D). This growth pattern is characteristic of a classic emulsion mechanism. Importantly, these results were achieved using only 2.3 wt % of SDS surfactant relative to the monomer. By maintaining high stability and controlled polymerization without prior deoxygenation and homogenization, this ab initio photoATRP offers a more sustainable and practical alternative to miniemulsion or microemulsion techniques that typically require high surfactant loadings or high-energy homogenization.

**Figure 1:**
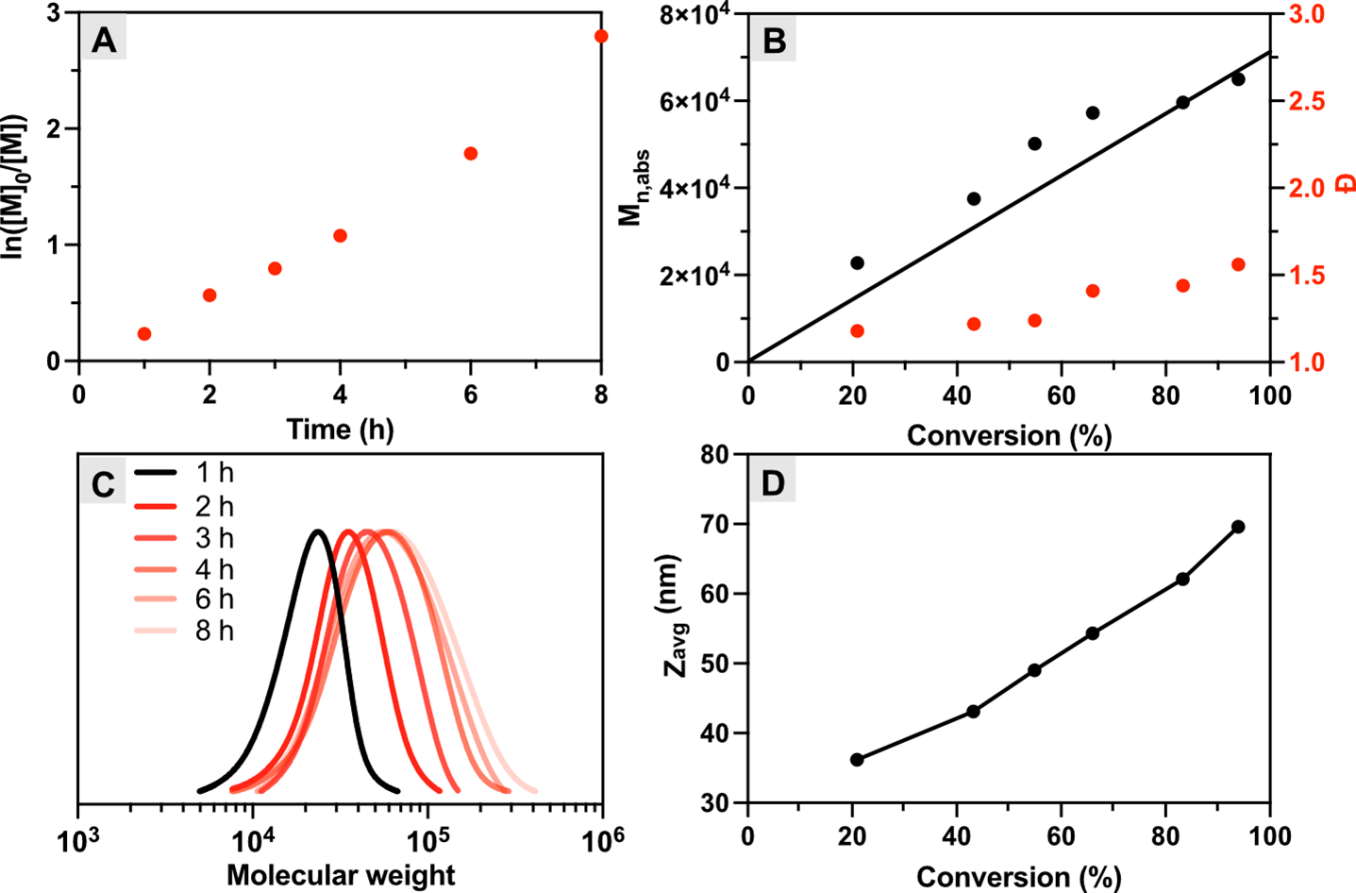
Ab initio emulsion photoATRP under NIR light. (A) First-order kinetic plot. (B) Evolution of molecular weights and molecular weight distributions with monomer conversion. (C) SEC traces and (D) particle size evolution over time. Reaction conditions: [BMA]/[HO-EBiB]/[MB^+^]/[CuBr_2_/TPMA (1:1 molar ratio)]/[TEOA] = 500/1/0.025/0.1/0.6, [M] = 20 vol % of the total, [SDS] = 2.3 wt % relative to BMA, [NaBr] = 0.1 M, irradiated under NIR LED (740 nm, 20 mW cm^–2^) in a one dram vial (diameter = 15 mm) with stirring.

### Chain Extension and Temporal Control

To further demonstrate the versatility and robust control of the ab initio emulsion ATRP, the chain-end fidelity of synthesized polymers was investigated. First, pBMA was synthesized under our typical classic emulsion conditions (*M*_n,app_ = 13,700, *Đ* = 1.20) and subsequently used as a macroinitiator for the chain extension of BMA. Compared with the pBMA macroinitiator, the SEC traces of the resulting pBMA-*b*-pBMA block copolymer show a clear shift to the high MW region (*M*_n,app_ = 72,000, *Đ* = 1.48) with a high conversion of 97%. This successful chain extension confirms the high degree of chain-end functionality retained during the ab initio emulsion process.

Temporal control over the polymerization enables modulation of heat transfer, which could be a critical requirement for large-scale emulsion polymerization in industrial settings.^19^ Taking advantage of photoinduced polymerization, facile temporal control was achieved by switching the light on and off (Figure 2B). Notably, polymerization proceeded exclusively under the irradiation of red light. When the light was off, negligible monomer conversion was observed (Table S2). Polymerization resumed upon irradiation with NIR light, facilitated by the photoinduced regeneration of the ATRP activator via excited MB^+^. The alternating cycles of NIR light on and off were repeated several times, demonstrating excellent temporal control. The resulting polymer showed good agreement between the experimental and theoretical values (*M*_n,abs_ = 88,600, *M*_n,th_ = 60,900) with low dispersity (*Đ* = 1.20).

**Figure 2:**
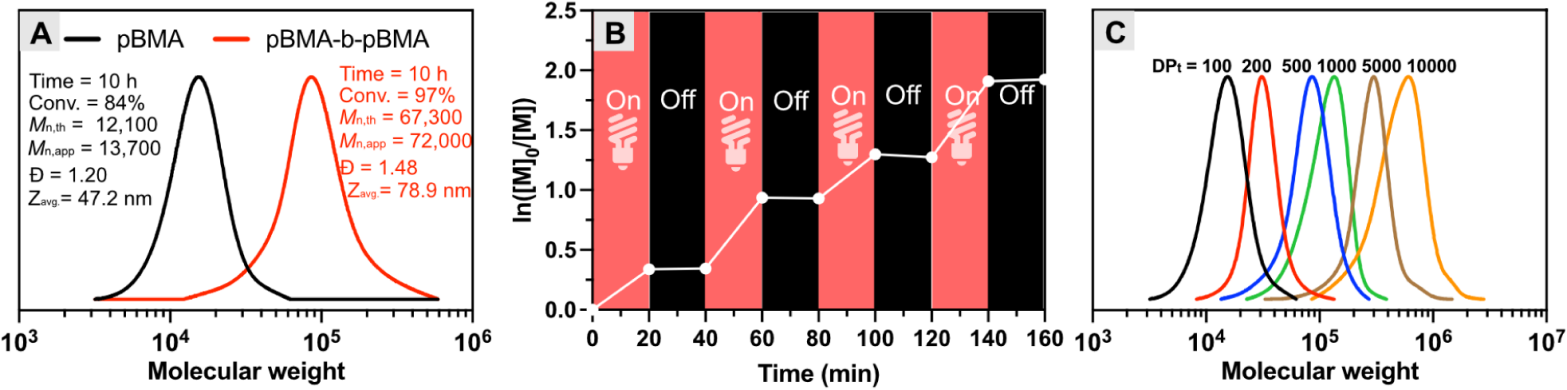
(A) Chain extension of pBMA macroinitiator with BMA. (B) Temporal control under NIR light. (C) SEC traces of pBMA with different DP_T_ by ab initio photoATRP.

### Tailoring Targeted Degrees of Polymerization

Emulsion polymerization offers distinct advantages over homogeneous systems for producing high MW polymers, primarily due to the reduced chain termination in confined spaces and lower viscosity in dispersed media. However, conventional FRP in emulsion is often limited to producing high MW with poor control and cannot precisely tune the MW, which significantly limits the applications of its products. To address these limitations, precise engineering of molecular weights was demonstrated by varying the targeted degree of polymerization (DP_T_) from 100 to 10,000. As shown in Table 2 and Figure 2C, the polymerization maintained good control across this broad range with high conversion (>60%) and low dispersity (1.13≤ *Đ* ≤ 1.29). The *M*_n,abs_ of the resulting polymers showed good agreement with theoretical values for DP_T_ from 100 to 5,000. The SEC traces for the resulting polymers remained monomodal and symmetric across these varying DPs. However, a more pronounced MW deviation from *M*_n,th_ at high DP_T_ = 10,000 was observed, likely due to an increased percentage of newly formed chains during dual catalysis, as the ATRP initiator concentration decreased at high DP_T_.^43^

**Table 2:** Synthesis of pBMA with Varying DP_T_ ^a^

Entry	DP_T_	Time (h)	Conv. (%)^b^	*M*_n,th_	*M*_n,app_ ^c^		**Đ* ^c^*
1	100	10	83.8	12,100	13,700	14,500	1.20
2	200	10	84.0	24,100	29,800	31,90031,900^d^	1.13
3	500	10	95.3	68,000	66,200	72,100	1.25
4	1000	10	99.0	141,000	136,000	150,400	1.29
5	5,000	10	60.2	428,600	258,800	334,000	1.18
6	10,000	22.5	72.6	1,032,200	438,200	596,000	1.29

a Reaction conditions: [BMA] = 20 vol % of the total, [SDS] = 6.9 wt % relative to BMA, [NaBr] = 0.1 M, irradiated under red LED (640 nm, 25 mW cm^–2^) in a one dram vial with stirring. Exact molar ratios of [BMA]/[HO-EBiB]/[MB^+^]/[CuBr_2_/TPMA (1:1 molar ratio)]/[TEOA] for each DP_T_ are provided in the Supporting Information.

b The monomer conversion is determined by gravimetric analysis.

c Molecular weight (*M*_n,app_) and dispersity (*Đ*) were determined by SEC analysis (THF as eluent) calibrated to polystyrene standards.

d The absolute molecular weight (*M*_n,abs_) was determined by Mark–Houwink calibration.

### Polymerization in Flask (25 mL)

To assess the scalability and practical applicability of ab initio emulsion photoATRP, the reaction was scaled up from a one dram vial (4 mL) to a round-bottomed flask (RBF, 25 mL). The polymerization in the RBF proceeded efficiently, reaching 78% monomer conversion within 2.5 h (Figure 3). The resulting polymer exhibited good control over molecular weight (*M*_n,app_ = 72,900, *M*_n,th_ = 55,500) while maintaining a low dispersity of 1.21. Furthermore, the resulting latex had an average particle size of 47.2 nm, which is in good agreement with the particle sizes obtained in a small vial (entry 1, Table 1). This successful scale-up in the RBF highlights the potential of this NIR-driven ab initio emulsion for larger-scale polymer production with precise structural control.

**Figure 3:**
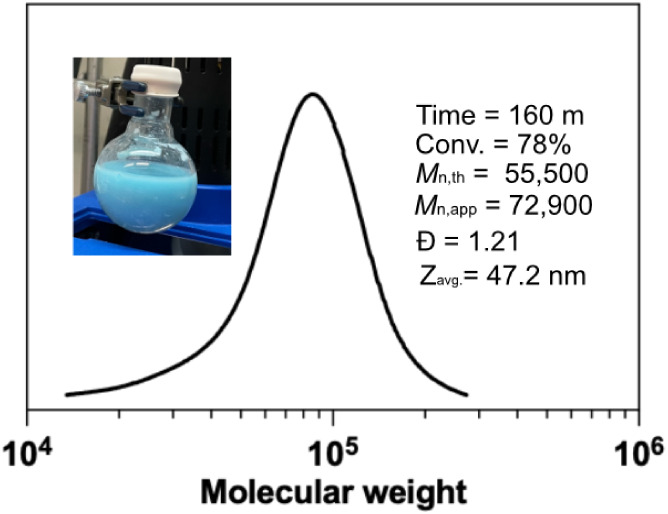
Ab initio emulsion photoATRP kinetics in an RBF (25 mL). Reaction conditions: [BMA]/[HO-EBiB]/[MB^+^]/[CuBr_2_/TPMA (1:1 molar ratio)]/[TEOA] = 500/1/0.025/0.1/0.6, [M] = 20 vol % of the total, [SDS] = 6.9 wt % relative to BMA, [NaBr] = 0.1 M, irradiated under red LED (640 nm, 25 mW cm^–2^) in an RBF under stirring.

## Conclusions

In conclusion, this work established the first O_2_-tolerant ab initio emulsion photoATRP system, operating under red and NIR light without prior deoxygenation or homogenization at surfactant loadings as low as 2.3 wt % relative to the monomer. This discovery was facilitated by dual catalysis using the PC methylene blue (MB^+^), the interfacial ion-pair catalyst [X–Cu^II^L^+^/DS^–^], and a water-soluble initiator (HO-EBiB) for mediating controlled polymerization via a classic emulsion mechanism. The proposed mechanism involves the reductive quenching of the excited MB^+^ by the water-soluble ED (TEOA), forming the MB radical. Subsequently, the MB radical reduces [X-Cu^II^/L]^+^ to [Cu^I^/L]^+^. The resulting ATRP activator reacts with the hydrophilic initiator HO-EBiB to initiate polymerization in the aqueous phase. The formed oligomers are captured by micelles, in which polymerization continues to eventually generate a stable emulsion. Kinetic studies, chain extension experiments, and the synthesis of polymers with varying degrees (DP_T_ = 100 to 10,000) confirmed the well-controlled and versatile nature of our approach. Most importantly, well-controlled polymerization was achieved without prior homogenization and deoxygenation at surfactant loadings as low as 2.3 wt %, substantially lower than the quantities required for miniemulsion or microemulsion RDRP. Overall, the long-wavelength light-driven ab initio emulsion ATRP offers a promising approach for the facile and precise synthesis of polymer materials.

## Supplementary Material



## Notes

Additional details of experimental procedures and experimental results, including digital camera images of the reaction setup, SEC traces, DLS, and temporal control results (PDF)
