# Supplementary material for: Oxygen-Tolerant Ab Initio Emulsion ATRP Driven by Red or Near-Infrared Light
Source: Macromolecules. 2026 Jun 22;59(13):7541–6. doi: 10.1021/acs.macromol.6c01379 (PMC13374375; doi:10.1021/acs.macromol.6c01379)
Supplement: Supplementary file 1 [file ma6c01379_si_001.pdf]

Supporting Information

Oxygen-Tolerant Ab Initio Emulsion ATRP Driven by Red or Near-Infrared Light

Xiaolei Hu, Kangping Liu, Krzysztof Matyjaszewski*
Department of Chemistry, Carnegie Mellon University, Pittsburgh, Pennsylvania 15213, USA
K.M. (km3b@andrew.cmu.edu)

Table of Contents

Materials and Instruments **3**

Materials.. 3

Instruments.. 3

Experimental Procedures.. **3**

General procedure for ab initio photoATRP of BMA 3

Synthesis of pBMA with varying DP_T.. 4

Chain extension 4

Temporal control 4

References **8**

Materials and Instruments

Materials

Unless otherwise noted, all chemicals were purchased from commercial sources and used as received. Methylene blue (MB^+, 99%), n-butyl methacrylate (BMA, 99%), copper(II) bromide ($CuBr_2$, 99.99%), 2-hydroxyethyl α-bromoisobutyrate (HO-EBiB, 95%), sodium bromide (NaBr, ≥99%), triethanolamine (TEOA, ≥99%), sodium dodecyl sulfate (SDS, 99%), were purchased from *Sigma-Aldrich*. BMA was passed through a column of basic alumina to remove the inhibitor before use. Tris(2-pyridylmethyl)amine (TPMA, 99%) was purchased from *AmBeed*. Water (HPLC grade) and tetrahydrofuran (THF, HPLC grade) were purchased from *Fisher Chemical*.

Instruments

Polymerization was conducted in a EvoluChem™ PhotoRedOx Box purchased from *Hepatochem* with varying LEDs. LED with red light (640 nm, 25 mW cm^{-2}) was purchased from *Kessil*; NIR light LED (740 nm, 20 mW cm^{-2}) was purchased from *Hepatochem.* Monomer conversion during classic emulsion photoATRP was determined by gravimetric analysis after evaporating the samples at 110 °C for at least 1 h. SEC measurements of pBMA were conducted using *PSS* columns (Styrogel 10^2, 10^3, 10^4, 10^5 Å) with THF as the eluent at 35 °C and the flow rate of 1 mL/min. Linear poly(methyl methacrylate) standards were used for SEC calibration. Absolute molecular weight ($M_{n,abs}$) was determined by Mark-Houwink calibration with K and a value of PS and pBMA from the literature.[1, 2] Particle sizes were determined by using a Zetasizer Nano from *Malvern Instruments, Ltd*.

Experimental Procedures

General procedure for ab initio photoATRP of BMA

Stock solutions of HO-EBiB (100 mM in H_2O), $CuBr_2$/TPMA complex (1:1 molar ratio, 50 mM in H_2O), MB^+ (1.88 mM in H_2O), TEOA (100 mM in H_2O), NaBr (2 M in H_2O), and SDS (300 mM in H_2O) were prepared. A typical aqueous phase mixture was then prepared by mixing HO-EBiB stock (100.6 µL), MB^+ stock (134.2 µL), $CuBr_2$/TPMA stock (20.1 µL), TEOA stock (60.39 µL), NaBr stock (200 µL), SDS stock (571 µL), and H_2O (2.11 mL) (total volume = 3.5 mL). The prepared aqueous mixture was transferred to the one-dram vial (diameter = 15 mm) equipped with a magnetic stirring. BMA (800 µL) was then added to top of aqueous mixture in the vial. The final concentrations were BMA (1.26 M), MB^+ (62.9 µM), $CuBr_2$/TPMA (0.25 mM), HO-EBiB (2.52 mM), TEOA (1.51 mM), and SDS (6.9 wt%). The polymerization mixtures were irradiated under red light LEDs (640 nm, 25 mW cm^{-2}) under stirring (700 rpm) in the EvoluChem™ PhotoRedOx Box. Samples were withdrawn periodically during the polymerization for gravimetric analysis and SEC measurement.

Supporting Information

Synthesis of pBMA with varying DP_T

The target degree of polymerization (DP_T = 100-10,000) was varied by adjusting the [BMA]:[HO-EBiB] ratio while [BMA] was held constant at 1.26 M (exact molar ratios for each DP_T are listed below). Classic emulsion photoATRP mixtures then were prepared following the general procedure and transferred to a one-dram vial (diameter = 15 mm) equipped with a magnetic stirring bar. Polymerizations were conducted under red LED irradiation (640 nm, 25 mW cm^{-2}) under stirring. At the end of the polymerizations, samples were collected for gravimetric analysis and SEC analysis.

Reaction conditions ([BMA]/[HO-EBiB]/[MB^+]/[$CuBr_2$/TPMA]/[TEOA]):

DP_T = 100/1/0.05/0.02/0.12

DP_T = 200/1/0.01/0.04/0.24

DP_T = 500/1/0.025/0.1/0.6

DP_T =1000/1/0.025/0.1/0.6

DP_T = 5000/1/0.025/0.3/0.6

DP_T =10000/1/0.1/0.6/1.2

Chain extension

For the synthesis of pBMA-*b*-pBMA block copolymer, pBMA with DP_T = 100 was first synthesized using molar ratios of [BMA]/[HO-EBiB]/[MB^+]/[$CuBr_2$/TPMA]/[TEOA] = 100/1/0.005/0.02/0.12 and irradiated by red LED (640 nm, 25 mW cm^{-2}) under stirring (700 rpm). The crude macroinitiator pBMA obtained directly after polymerization was then used without purification to prepare the ATRP mixture for chain extension with BMA at DP_T = 400 under [BMA]/[pBMA]/[MB^+]/[$CuBr_2$/TPMA]/[TEOA] = 400/1/0.02/0.08/0.48. The polymerization mixture was irradiated under red LED irradiation (640 nm, 25 mW cm^{-2}) under stirring. Finally, the sample was withdrawn for gravimetric analysis and SEC measurement.

Temporal control

The classic emulsion ATRP mixture was prepared according to the general procedure at [BMA]/[HO-EBiB]/[MB^+]/[$CuBr_2$/TPMA]/[TEOA] = 500/1/0.025/0.1/0.6. The polymerization mixtures were irradiated under red LED (640 nm, 25 mW cm^{-2}) under stirring. The light was switched on/off periodically every 20 min and aliquots of samples were withdrawn at each time point for gravimetric analysis and SEC measurement.

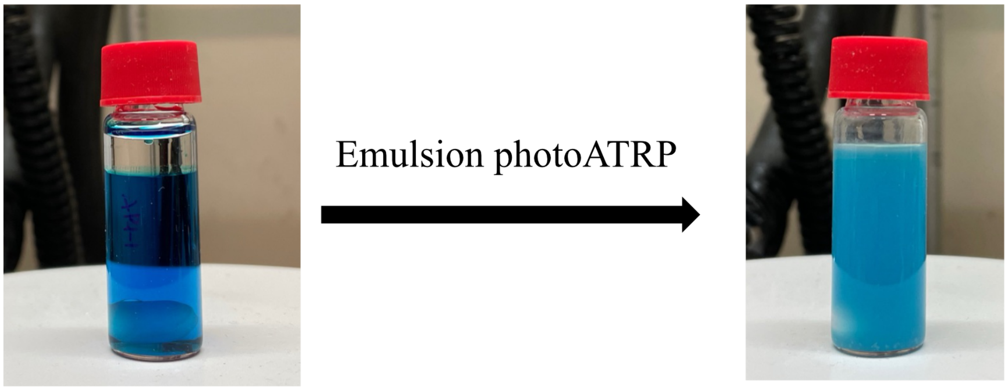

Figure S1. Digital camera images demonstrating the ab initio emulsion photoATRP before and after polymerization.

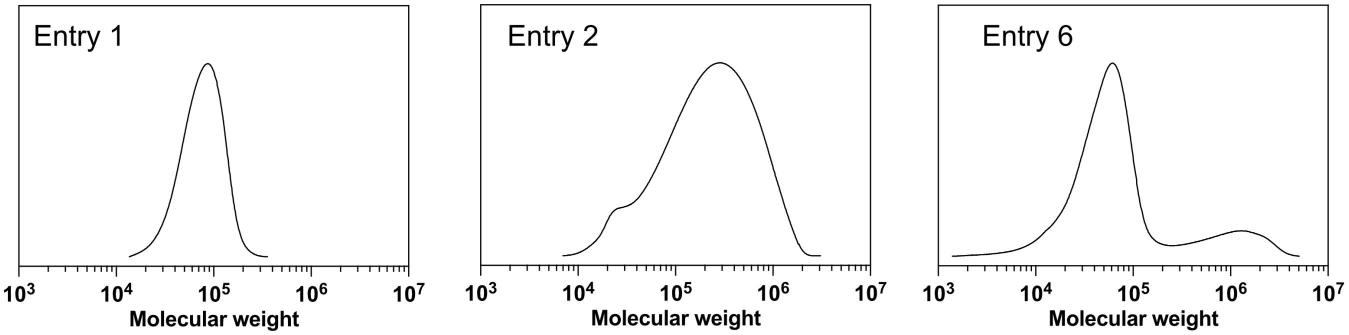

Figure S2. SEC traces for the ab initio emulsion photoATRP results shown in Table 1.

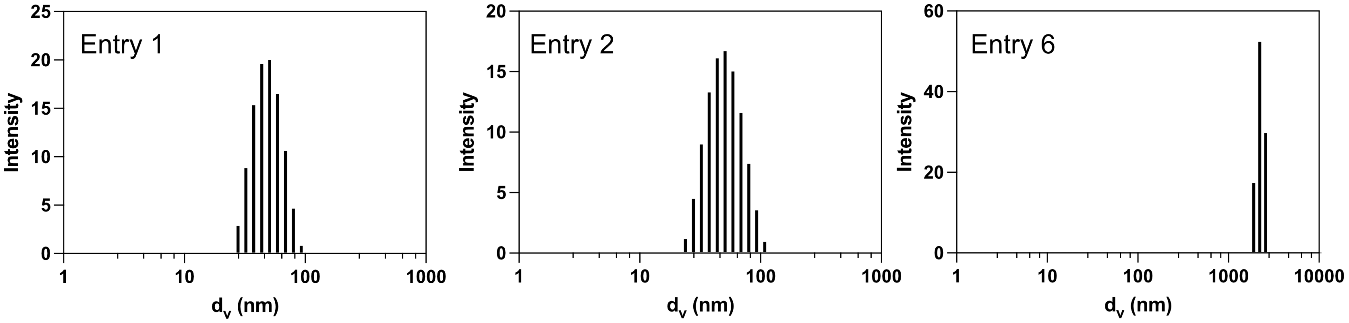

Figure S3. DLS results for ab initio emulsion photoATRP results shown in Table 1.

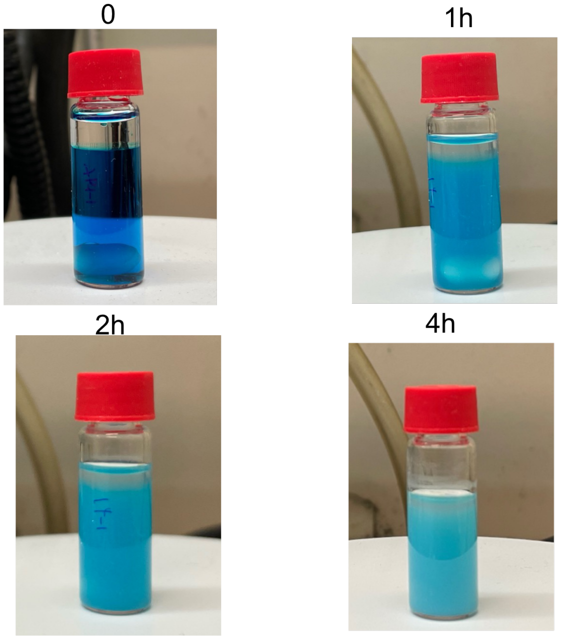

Figure S4. Digital camera image of ab initio emulsion photoATRP "cocktail" in a one-dram vial (diameter = 15 mm) with polymerization progressing. (Noting that samples were withdrawn for kinetic analysis)

Table S1. Ab initio emulsion photoATRP under varying SDS.[a]

Entry	SDS	Time (h)	Conv. (%)[b]	$M_{n,th}$	$M_{n,app}$[c]	$M_{n,abs}$[c]	Đ[c]	$M_{n,abs}$[d]
1	6.9	4.5	95.3	68,000	66,200	72,100	1.25	45.3±0.1
2	4.6	10	73.3	52,400	69,700	76,000	1.31	61.0±0.2
3	2.3	6	82.2	58,600	65,400	71,200	1.37	64.0±0.1

Reaction conditions: [BMA]/[HO-EBiB]/[MB^+]/[$CuBr_2$/TPMA (1:1 molar ratio)]/[TEOA] = 500/1/0.025/0.1/0.6, [M] = 20 vol% to total, [SDS] = x wt% relative to BMA, [NaBr] = 0.1 M, irradiated under red LED (640 nm, 25 mW cm^{-2}) in a one-dram vial with stirring.

Table S2. Temporal control over ab initio photoATRP of BMA under NIR light.[a]

Entry	Time (min)	Light	Conv. (%)
1	0	OFF	0
2	0-20	ON	28.7
3	20-40	OFF	29.1
4	40-60	ON	60.8
5	60-80	OFF	60.6
6	80-100	ON	72.7
7	100-120	OFF	72.0
8	120-140	ON	85.2
9	140-160	OFF	85.4

Reaction conditions: [BMA]/[HO-EBiB]/[MB^+]/[$CuBr_2$/TPMA (1:1 molar ratio)]/[TEOA] = 500/1/0.025/0.1/0.6, [M] = 20 vol% to total, [SDS] = 6.9 wt% relative to BMA, [NaBr] = 0.1M, irradiated under NIR LED (740 nm, 20 mW cm^{-2}) in a one-dram vial with stirring.

References

1. Spatorico, A. L.; Coulter, B., Molecular weight determinations by gel-permeation chromatography and viscometry. *Journal of Polymer Science: Polymer Physics Edition* **1973,** *11* (6), 1139-1150.
2. Gruendling, T.; Junkers, T.; Guilhaus, M.; Barner-Kowollik, C., Mark–Houwink Parameters for the Universal Calibration of Acrylate, Methacrylate and Vinyl Acetate Polymers Determined by Online Size-Exclusion Chromatography—Mass Spectrometry. *Macromol. Chem. Phys.* **2010,** *211* (5), 520-528.
